# Triage, Surge Capacity, and Epidemic Emergency Unit: An experience from the 2019 dengue outbreak at a Tertiary Care Centre

**DOI:** 10.31729/jnma.4771

**Published:** 2020-04-30

**Authors:** Ashis Shrestha, Sumana Bajracharya, Darlene Rose House

**Affiliations:** 1 Department of General Practice and Emergency Medicine, Patan Academy of Health Sciences, Lagankhel, Nepal; 2 Department of Paediatric Emergency, Indiana University

**Keywords:** *dengue*, *epidemics*, *surge capacity*, *triage*

## Abstract

In September 2019, a dengue outbreak was observed in many non-endemic areas of Nepal. The emergency department of Patan Academy of Health Sciences also received febrile patients exceeding usual daily numbers. This surge of the patient was managed by epidemic triage and activation of surge capacity. A part of the surge plan was to activate an epidemic emergency unit. An observation ward adjacent to the emergency was used for the epidemic emergency unit. The febrile patients who were triaged yellow and green were treated in this unit. The patients who were triaged as Red were treated in the emergency department.

## INTRODUCTION

In September 2019, a dengue outbreak was observed in many non-endemic areas of Nepal.^1^ Patan Hospital, which is a tertiary care hospital in Kathmandu valley, Nepal saw 2567 cases of febrile illness in that month. This was 23.8% of the total patient visiting in that month, out of which the dengue screening test was positive in 658 (25.6%) patients. To manage this influx of patients, outbreak response activities were carried out. This viewpoint describes the experience of triage, surge capacity, and epidemic emergency units at Patan Academy of Health Sciences, Nepal, during dengue outbreak 2019.

### Triage

Triage is an essential component of disaster that helps to minimize morbidity and mortality.^2^ Triage during a disaster is different than during-disaster,^3^ moreover there is a difference in triage process when the incidence is trauma versus infectious disease outbreak. During an outbreak, triage depends on the type of outbreak, its communicable potential and the level of safety precaution that is required.^4^ Since an outbreak may last for a long period with variable numbers of patients coming per day, there might be some overlap between regular triage and outbreak triage.

**Figure 1.**
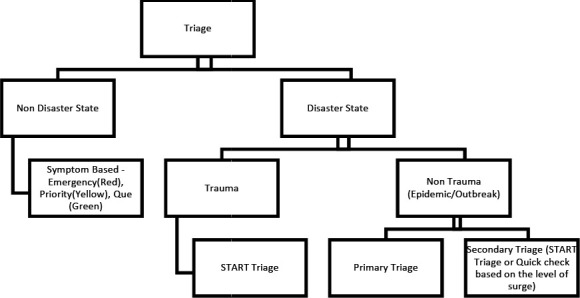
Triage during a disaster and non-disaster state.

At Patan Academy of Health Sciences during the non-disaster period quick check and emergency treatment of adolescents and adults are used for triaging.^5^ However, during a disaster, Simple Triaging and Rapid Treatment (START)^6^ is used (Figure 1).

In the case of an epidemic outbreak, primary triage and secondary triaging are done. Primary triage consists of a screening question (i.e. Do you have a fever, headache, retro-orbital pain?). In Secondary triage START or Quick Check triaging is done based on the level of the surge.

During a dengue outbreak this year (2019), there was an increasing number of febrile patients visiting the emergency department. We did screening triage with a screening question; “Do you have fever, headache, retro-orbital pain?” followed by quick check triage. All patients coming to the emergency were asked screening questions. Patients with positive screening questions and not requiring resuscitation were sent to the Epidemic Emergency unit (EEU). Primary and secondary triage was done from the same desk. The separate area for primary and secondary triage is important only if the outbreak is highly communicable requiring safety precaution more than standard precaution or number of patients exceeding the capacity to manage. In secondary triage, START triage can be done if the surge of the patient is very high.

### Surge Capacity

**Figure 2.**
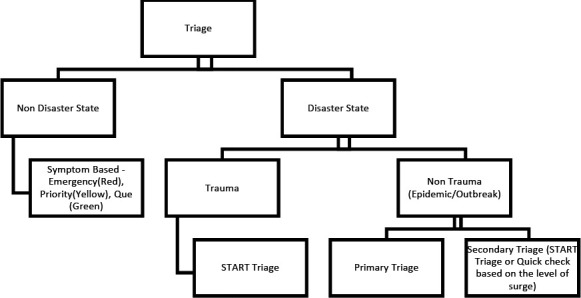
Surge planning during the outbreak.

Surge needs to be activated if the influx of patients exceeds the capacity of the hospital to manage it with the available resources. Activating surge during disease outbreak depends on multiple factors like the communicable potential of disease, case fatality rate, and available resources. There is no clear demarcation to activate surge, we defined surge based on its communicable potential (Figure 2).

If the disease is highly communicable even a single case may be very much resource consuming, so it needs surge activation which we defined as surge 1.

During this outbreak, the number of patients in the emergency department increased to 300 patients per day which would otherwise have been 150 patients per day on average. This number of the patient included patients with suspected and probable dengue and patients with other emergency conditions. With the increased volume, we activated surge 1 with the addition of extra manpower. However, since this was not sufficient, we activated the surge 2 plan which is the extension of emergency to the EEU. An observation unit adjacent to the emergency was converted to EEU. Activation and scale-up of the surge plan were based on daily debriefing and department's consensus.

### Epidemic Emergency Unit

Available space near an emergency or ward can be used to make EEU. This will divert the infectious patient due to an outbreak from regular emergency patients. In the case of highly communicable disease, this unit needs to be isolated, requires trained manpower and requires huge resources.

We started EEU as an extension of an emergency. An observation ward adjacent to the emergency was used for this purpose. Any patient with a positive screening question and not requiring resuscitation were sent to this unit. It ran 12 hours a day from morning 8 am to 8 pm. The EEU was a nine-bed ward with one medical officer and one nursing to cover the duty hours. The unit was equipped with the ability to do draw blood for laboratory testing, administer intravenous fluids, and provide medications to patients. A treatment protocol was made available for the management of patients. The patient was then either discharged or admitted to the ward.

## DISCUSSION

We found that proper management of triage and epidemic outbreak units is key to the management of a huge influx of patients in the emergency. During the influx of patients, there is a massive demand for medical assistance while the hospital will be operating at its maximum capacity, resulting in long waiting lines, restricted access to healthcare services, and a shortage of hospital beds.^7,8^ In such circumstances, patients who could have been diagnosed and treated at an earlier stage, using low-complexity diagnostic and therapeutic tools, may progress to more severe forms of the disease.^9^

We found an epidemic outbreak unit useful during such outbreaks along with a written plan to scale it up and down. In our experience, EEU helped us to manage the crowd, which would otherwise have been in an emergency. We felt that this is very essential as the influx of patients will last for many days. We found this approach simple, low-cost, and effective as it used available resources in the care of patients. A similar concept was used in the form of hydration tent in Brazil in 2008 and showed a decreasing number of hospitalization.^10^ However, it is not very clear to what is the cut off number of the patient at which we scale up the plan. In our experience, the cut off number is not feasible to scale up the plan, the only thing that might be helpful is daily debriefing, gathering the local information, discussing in the team and deciding.

## CONCLUSIONS

Implementing triage, establishing surge capacity and provision of EEU was a simple, low-cost, and effective approach to manage patients during a dengue outbreak.

## Conflict of Interest

**None.**
